# Rigorous Assessment of Cl^−^‐Based Anolytes on Electrochemical Ammonia Synthesis

**DOI:** 10.1002/advs.202204205

**Published:** 2022-10-17

**Authors:** Zengxiang Lv, Leiduan Hao, Zhibo Yao, Weixiang Li, Alex W. Robertson, Zhenyu Sun

**Affiliations:** ^1^ State Key Laboratory of Organic‐Inorganic Composites Beijing University of Chemical Technology Beijing 100029 P. R. China; ^2^ Department of Physics University of Warwick Coventry CV4 7AL UK

**Keywords:** ammonium oxidation, electrochemical ammonia synthesis, crossover of ammonium, anodic Cl^−^ oxidation

## Abstract

Many challenges in the electrochemical synthesis of ammonia have been recognized with most effort focused on delineating false positives resulting from unidentified sources of nitrogen. However, the influence of oxidizing anolytes on the crossover and oxidization of ammonium during the electrolysis reaction remains unexplored. Here it is reported that the use of analytes containing halide ions (Cl^−^ and Br^−^) can rapidly convert the ammonium into N_2_, which further intensifies the crossover of ammonium. Moreover, the extent of migration and oxidation of ammonium is found to be closely associated with external factors, such as applied potentials and the concentration of Cl^−^. These findings demonstrate the profound impact of oxidizing anolytes on the electrochemical synthesis of ammonia. Based on these results, many prior reported ammonia yield rates are calibrated. This work emphasizes the significance of avoiding selection of anolytes that can oxidize ammonium, which is believed to promote further progress in electrochemical nitrogen fixation.

## Graphical abstract

It is reported that the use of analytes containing halide ions (Cl^−^ and Br^−^) can rapidly convert the ammonium into N_2_, which further intensifies the crossover of ammonium. This study emphasizes the significance of avoiding selection of anolytes that can oxidize ammonium during N_2_ electrolysis.

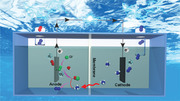

## 1 Introduction

Ammonia (NH_3_) is one of the most vital basic chemical materials, as it is extensively used across industry, agriculture, pharmaceutical, and energy storage. Currently, the industrial synthesis of ammonia is mainly dependent on the Haber–Bosch (HB) process, which requires harsh conditions (300–500 °C, 50–350 atm) and thus causes tremendous energy consumption and excessive CO_2_ emission.^[^ ^1^, ^2^, ^3^ ^]^ To ameliorate these adverse impacts, synthesis of ammonia by the electrochemical N_2_ reduction reaction (ENRR), which can be driven by renewably generated electricity, has recently attracted heightened research attention, and is considered a promising alternative to the HB process.^[^ ^4^, ^5^, ^6^, ^7^ ^]^ Although massive efforts have been devoted to this emerging field, the ENRR is still plagued with limited activity and a multitude of other underlying problems. Over the past few years, a plethora of materials have been investigated for ENRR activity to yield NH_3_. Unfortunately, the quantities of NH_3_ produced in these reports are extremely low (usually in the ppm range or even much lower). This renders it difficult to confirm whether the NH_3_ evolved originates from N_2_ reduction or spurious nitrogen sources, which has hindered further development of ENRR.^[^ ^8^, ^9^, ^10^ ^]^

Previous works have reported a wide range of possible sources of false positive results in experiments, which has brought about considerable controversy in evaluating the ENRR performance. Confounding NH_3_ contamination can come from the air, human breath, glassware, and laboratory equipment, which makes the origin and quantification of NH_3_ extremely ambiguous.^[^ ^11^, ^12^, ^13^ ^]^ And during the reaction itself, NH_3_ generated from electrolysis could instead be attributed to the reduction of nitrogen‐containing compounds (for example, NO_3_^−^, NO_2_^−^, and NO*_x_*) that are typically present in the reactant gases, feedstock, and electrolyte, which are more easily reduced than N_2_.^[^ ^14^, ^15^ ^]^ In addition, some nitrogen‐containing catalysts have been proven to be readily reduced to NH_3_ during electrolysis^[^ ^16^ ^]^ owing to the easier activation of nitrogen in the lattice.^[^ ^17^ ^]^ Recent research has indicated that ammonium can traverse through the Nafion membrane into the anolyte during the ENRR experiments, which is clearly detrimental to the quantification of produced NH_3_.^[^ ^18^, ^19^ ^]^

Electrolytes are highly involved in electrocatalytic processes through interactions with catalyst surfaces, reactants, intermediates, and even products.^[^ ^20^, ^21^, ^22^, ^23^, ^24^ ^]^ However, previous work has only focused on the effect of the catholyte on the ENRR. The possible impact of the anolyte on the ENRR has not been seriously investigated thus far.

An H‐type electrolytic cell with an anode compartment and a cathode compartment separated by a proton exchange membrane (PEM) is widely used in the ENRR. Various acidic, neutral, and alkaline electrolytes have been screened and applied for ENRR,^[^ ^25^, ^26^, ^27^ ^]^ among which HCl solution has been commonly used since it can provide a sufficient proton source to facilitate the coupled electron/proton N_2_ reduction process. However, the effect of using an HCl solution as the anolyte on the ENRR has yet to be explored. Here, we report for the first time that ammonium produced at the cathode tends to migrate through the PEM and is rapidly oxidized in Cl^−^‐based aqueous anolyte. More importantly, the oxidation of ammonium can further intensify the crossover process. Such occurrence can dramatically affect the quantification of NH_3_ evolved, leading to a false measured NH_3_ yield. Based on this observation, we reconsider prior reported data on NH_3_ production rates obtained with Cl^−^‐based anolyte in H‐cells. Our finding can be also extended to other electrocatalytic reactions involving N‐containing reduction reactions (e.g., NO*_x_* reduction reaction) when using Cl^−^‐based analyte, thus providing guidance for the selection of suitable analyte during reduction reactions.

## 2 Results and Discussion

For an aqueous ENRR system, the reduction reactions of N_2_ and protons (or water) generally occur at the cathode while the oxygen evolution reaction (OER) takes place at the anode. However, when HCl solution is used as an anolyte, the reaction at the anode may be different from other aqueous electrolytes due to the presence of Cl^−^. As shown in Equations 1 and 2, the *E°* of the Cl_2_/Cl^−^ couple (1.36 V vs reversible hydrogen electrode (RHE)) is similar to that of the O_2_/H_2_O couple (1.23 V vs RHE). Considering that the oxidation of Cl^−^ to Cl_2_ only needs two electrons without the involvement of protons, it is mechanistically more favorable compared to the OER.^[^ ^28^ ^]^ Therefore, the oxidation of Cl^−^ may happen in parallel with the OER at the anode when HCl solution is used as the electrolyte

(1) $$2{\mathrm{Cl}}^{-}\to {\mathrm{Cl}}_{2}+2e^{-}E{}^{\circ }=1.36\;V\;\left(\mathrm{vs}\;\mathrm{RHE}\right)$$

(2) $$2H_{2}O\to O_{2}+4H^{+}+4e^{-}E^{\circ }=1.23\;V\;\left(\mathrm{vs}\;\mathrm{RHE}\right)$$

**Figure 1:**
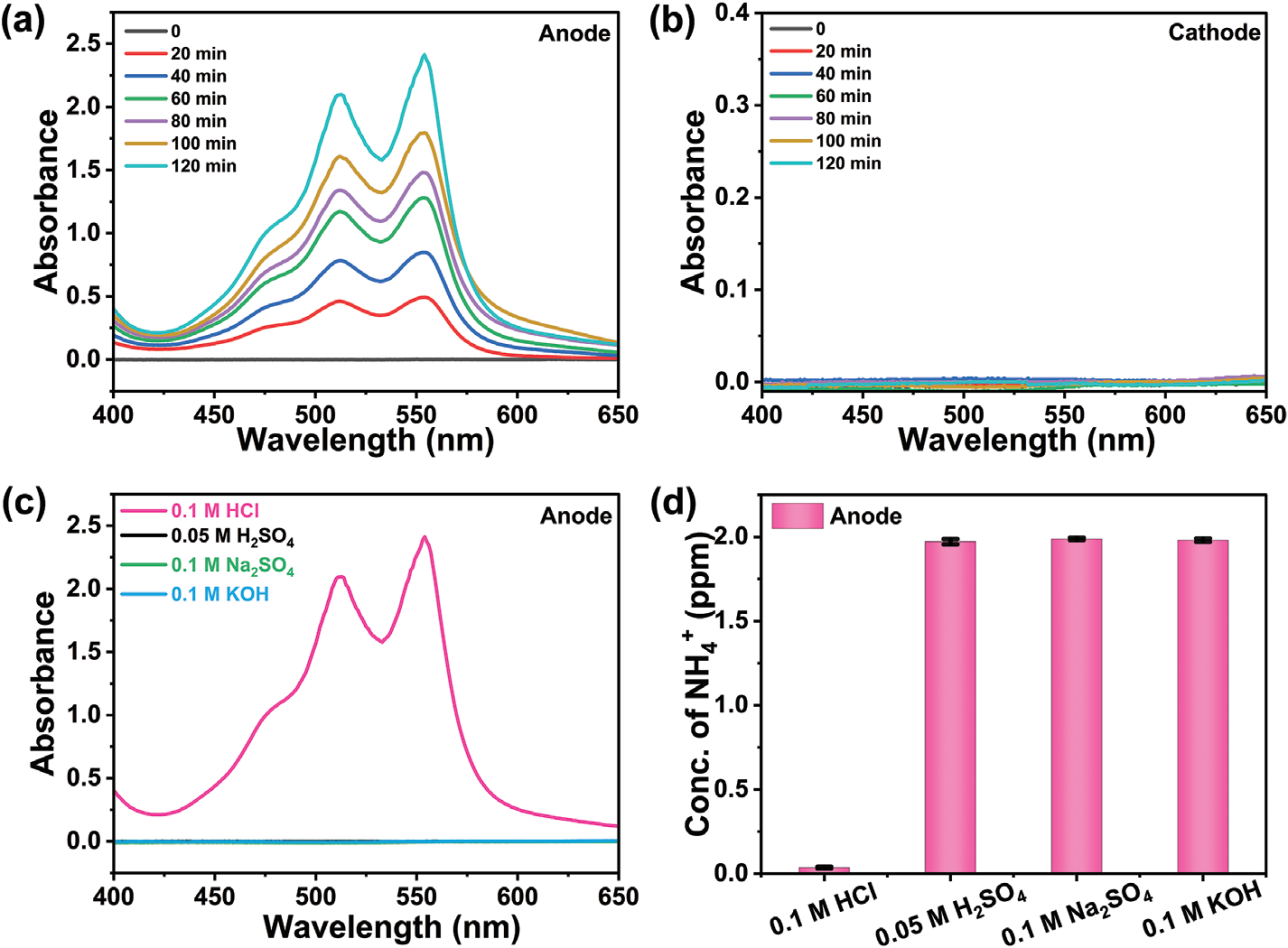
The UV–vis absorption spectra of a) Ar‐saturated 0.1 m HCl anolyte and b) Ar‐saturated 0.1 m HCl catholyte after electrolysis for different times at −0.4 V (vs RHE). c) The UV–vis spectra of different Ar‐saturated anolytes after 2 h of electrolysis at −0.4 V (vs RHE). d) Comparison of NH_4_^+^ concentration in different Ar‐saturated anolytes containing 2 ppm NH_4_^+^ after 2 h of electrolysis at −0.4 V (vs RHE).

To investigate whether the Cl^−^ would be oxidized during the ENRR, we designed electrolysis experiments using 0.1 m HCl solutions as both anolyte and catholyte in a typical H‐cell separated with a Nafion 117 membrane at a commonly reported potential (−0.4 V vs RHE). Since Cl_2_ can easily dissolve in water and form hypochlorous acid (HClO) (Equation 3), the amount of Cl_2_ from Cl^−^ oxidation was evaluated by the quantity of HClO.^[^ ^29^ ^]^ After 20 min of electrolysis, an HClO signal was clearly observed in the anolyte from the UV–vis spectra (**Figure** **1**a). The HClO accumulated with electrolysis time. No HClO was detected in the catholyte (Figure 1b). When using other electrolytes instead of HCl solution, including 0.05  H_2_SO_4_, 0.1m Na_2_SO_4_, and 0.1 m KOH, HClO was not found in the anolyte after 2 h of electrolysis, as illustrated in Figure 1c. These results provide excellent evidence that the oxidation of Cl^−^ happened at the anode when HCl was used as the anolyte. The generated HClO is a strong oxidizing agent, which is predicted to oxidize NH_4_^+^ (Equation 4).^[^ ^29^, ^30^ ^]^ To verify this, we conducted electrolysis experiments using various electrolytes containing 2 ppm of NH_4_^+^. It can be clearly seen from Figure 1d that nearly no NH_4_^+^ remained in the anode cell operated with 0.1 m HCl as an anolyte, while in other electrolytes without Cl^−^, the concentration of NH_4_^+^ did not change after electrolysis for 2 h.

(3) $${\mathrm{Cl}}_{2}+H_{2}O⇌\mathrm{HClO}+H^{+}+{\mathrm{Cl}}^{-}\left(pK_{a}=1.4\right)$$

(4) $$2{\mathrm{NH}}_{4}^{+}+3\mathrm{HClO}\to N_{2}+5H^{+}+3{\mathrm{Cl}}^{-}+3H_{2}O$$

**Figure 2:**
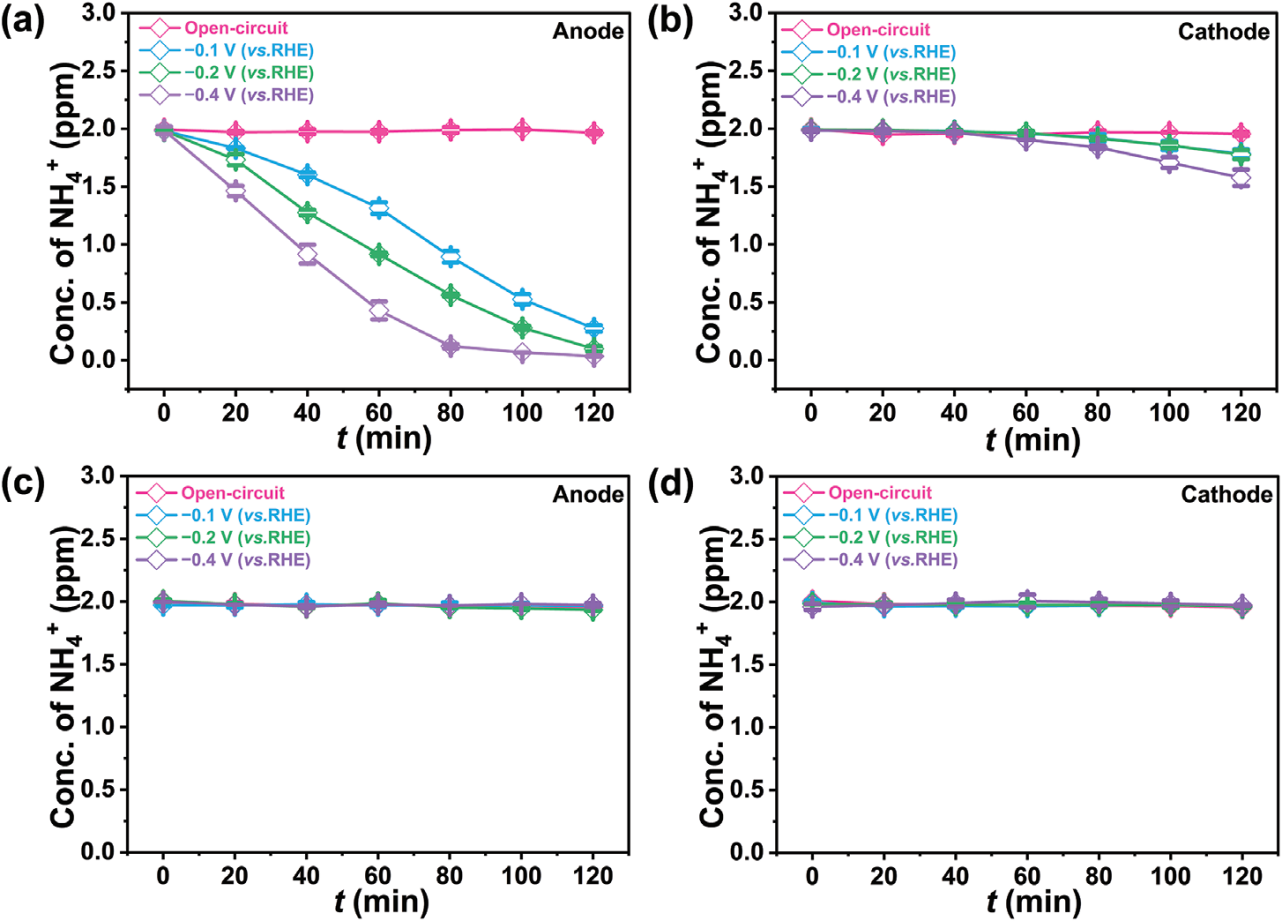
The evolution of NH_4_^+^ concentration in a) anolyte and b) catholyte which both contain 0.1 m HCl and 2 ppm NH_4_Cl as a function of electrolysis time at open circuit and various potentials. The concentration of NH_4_^+^ in c) anolyte and d) catholyte which both contain 0.05 m H_2_SO_4_ and 2 ppm NH_4_Cl versus electrolysis time at open circuit and various potentials.

To further explore the oxidation of NH_4_^+^ at the anode during electrolysis and its influence on the cathode, a series of experiments with 0.1 m HCl containing 2 ppm NH_4_^+^ as electrolytes for both anode and cathode cells were performed at open circuit and various potentials. The concentration of NH_4_^+^ in both cells remained almost unchanged with time at an open circuit potential (**Figure** **2**a,b). However, as we demonstrated above, when a threshold potential was applied, the concentration of NH_4_^+^ in the anode cell decreased rapidly, due to the electrochemical generation of oxidizing HClO. The rate of NH_4_^+^ oxidation increased concomitantly with overpotential, and approached zero with 2 h of electrolysis for all conditions studied. In regards to the cathode side, the concentration of NH_4_^+^ did not alter at the beginning of electrolysis. With prolonged electrolysis, the concentration difference between the two cells increased, leading to gradual migration of NH_4_^+^ from the cathode compartment to the anode side, followed by its oxidation. In particular, the concentration of NH_4_^+^ in the catholyte started to decrease after 40–60 min of electrolysis, indicating migration of NH_4_^+^ across to the anode cell, due to the NH_4_^+^ in the anolyte being almost completely oxidized within 80 min at −0.4 V (vs RHE). In addition to UV–vis spectroscopy, the Nessler reagent method and ion chromatography were combined to quantify the NH_4_^+^ concentration to ensure the accuracy of the experimental results (Figure S5, Supporting Information). In comparison, when 0.05 m H_2_SO_4_, 0.1 m Na_2_SO_4_, or 0.1 m KOH solution containing 2 ppm NH_4_^+^ were used as the electrolyte with the other conditions unchanged, the concentration of NH_4_^+^ in the two electrolytic cells remained constant throughout the whole electrolysis process (Figure 2c,d; Figures S6 and S7, Supporting Information). These results suggest that NH_4_^+^ at the anode side can be oxidized by the HClO generated from Cl^−^ oxidation under applied potentials, which caused the observed concentration difference between the two cells and intensified the subsequent crossover of NH_4_^+^ from the cathode to the anode side. Such an effect clearly brings about issues for the quantification of NH_3_ evolved during the ENRR. In the next step, we designed experiments to simulate the ENRR process and further identify the effect of NH_4_^+^ diffusion on the quantification of the reaction.

**Figure 3:**
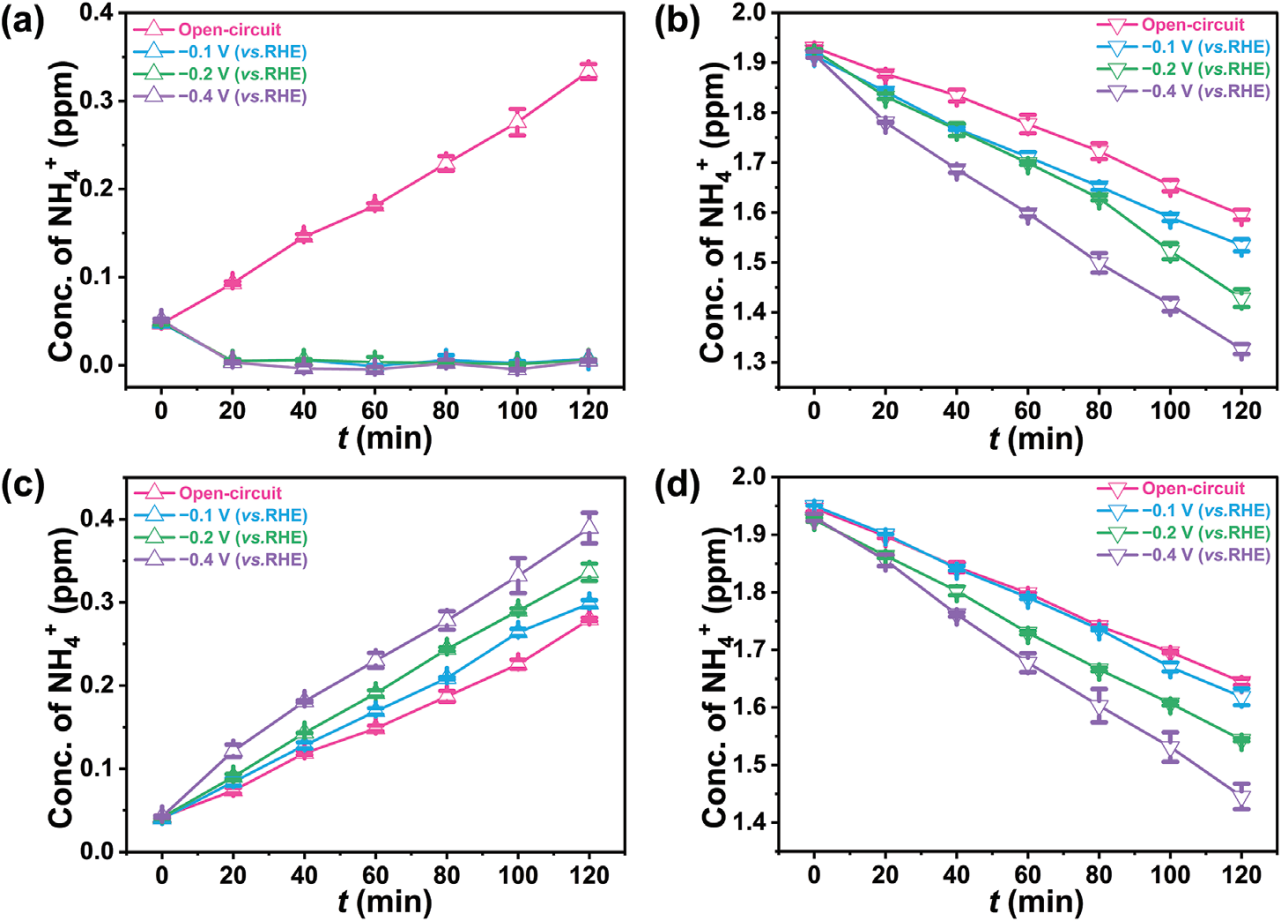
The concentration of NH_4_^+^ in 0.1 m HCl a) anolyte and b) catholyte versus time at open circuit and various potentials. The concentration of NH_4_^+^ in 0.05 M H_2_SO_4_ c) anolyte and d) catholyte versus time at open circuit and various potentials.

To witness the dynamic crossover of NH_4_^+^ from the catholyte to the anolyte followed by possible oxidation at the anode, we performed electrolysis experiments using 0.1 m HCl with added NH_4_^+^ as the catholyte, and bare 0.1 m HCl solution as the anolyte, mimicking the ENRR. Prior to the electrolysis, both catholyte and anolyte were purged with Ar for 20 min. Under open circuit, the level of NH_4_^+^ in the anolyte increased steadily with time, while it conversely decreased in the catholyte. This suggests the continuous migration of NH_4_^+^ from the catholyte to the anolyte through the membrane (**Figure** **3**a,b). After 120 min, 1.596 ppm of NH_4_^+^ was detected in the catholyte, accounting for 79.8% of the initial NH_4_^+^ amount in the catholyte. When a voltage was applied, the content of NH_4_^+^ in the catholyte dropped with a faster rate. However, distinct from the scenario occurring under open circuit, the amount of NH_4_^+^ in the anolyte did not rise, which was plausibly attributed to the oxidation of NH_4_^+^ (to N_2_) by the HClO electrochemically produced in the anolyte. The rate of decrease in NH_4_^+^ in the cathode cell was intensified with increasing overpotential. This can be explained by two processes: (1) the migration rate of ions in the solution increased with an enhanced electric field; (2) the oxidation rate of diffused NH_4_^+^ accelerated with more HClO generation under higher potentials, which in turn exacerbated the migration of NH_4_^+^ from the cathode to the anode cell. After 2 h of electrolysis at −0.4 V (vs RHE), only 1.32 ppm of NH_4_^+^ remained in the catholyte, ≈66% of the initial concentration. This strongly suggests that the NH_4_^+^ crossover would result in a significant confounding influence on the quantification of the produced ammonia on the cathode during ENRR. In addition to Nafion 117, similar phenomena were also observed using Nafion 115 and Nafion 211 proton exchange membranes for the electrolysis experiments (Figures S9 and S10, Supporting Information). When replacing 0.1 m HCl with 0.05 m H_2_SO_4_ under otherwise equivalent conditions, the NH_4_^+^ concentration exhibited similar behavior at open circuit (Figure 3c,d). At various applied potentials, the NH_4_^+^ concentration increased accordingly at the anode side, given that the diffused NH_4_^+^ was not oxidized in the 0.05 m H_2_SO_4_ electrolyte. The concentration of NH_4_^+^ in the cathode chamber decreased at slower rates compared with that seen in the 0.1 m HCl electrolyte. After 2 h of electrolysis at −0.4 V (vs RHE), a decrease of around 27.5% in NH_4_^+^ concentration took place in the catholyte, lower than that in the HCl case. Control experiments were also carried out with 0.1 m Na_2_SO_4_ and 0.1 m KOH as electrolyte, respectively. The results were similar to that with 0.05 m H_2_SO_4_. Furthermore, the oxidation of NH_4_^+^ in HCl anolyte was examined with a flow cell. The results indicated that the oxidation of NH_4_^+^ was more severe due to the crossover of products^[^ ^31^ ^]^ and the more vigorous reaction in flow cell reactors (Figure S13, Supporting Information). In order to probe whether there was NH_4_^+^ contamination during the operation, solutions without NH_4_^+^ were used as electrolytes for the same experiments. It could be clearly observed that almost no NH_4_^+^ was detected in both the anode and cathode cells after the reaction (Figure S14, Supporting Information), which demonstrated the reliability of our experimental results.

**Figure 4:**
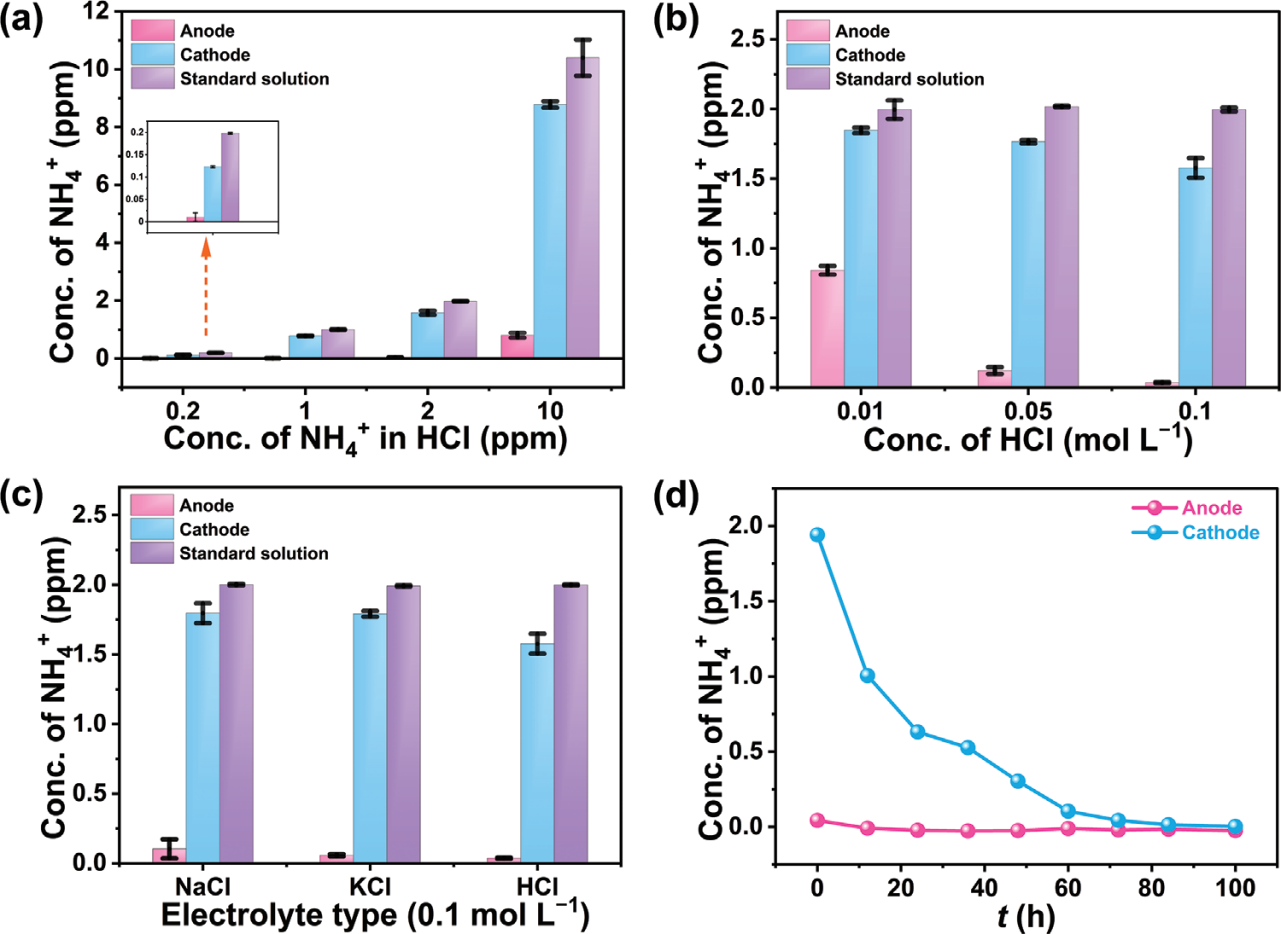
Comparison of NH_4_^+^ concentration after 2 h of electrolysis at −0.4 V (vs RHE) with a) 0.1 m HCl with diverse starting NH_4_^+^ concentrations (standard solution), b) varying HCl concentrations containing 2 ppm NH_4_^+^, and c) different Cl^−^‐based solutions containing 2 ppm NH_4_^+^. d) The evolution of NH_4_^+^ concentration in bare 0.1 m HCl anolyte and 0.1 m HCl catholyte containing 2 ppm NH_4_^+^ during 100 h of electrolysis at −0.4 V (vs RHE).

To gain more information about the diffusion and oxidation of NH_4_^+^, the following experiments were designed. (1) Various equivalent initial concentrations of NH_4_^+^ (0.2, 1, 2, and 10 ppm) in 0.1 m HCl were used as both catholyte and anolyte. After electrolysis for 2 h at −0.4 V (vs RHE), the NH_4_^+^ in the anode chamber was almost exhausted (**Figure** **4**a). Meanwhile, the NH_4_^+^ in the cathode chamber reduced by varying degrees owing to the diffusion of NH_4_^+^. Specially, since the NH_4_^+^ with lower concentrations (0.2, 1, and 2 ppm) was completely oxidized in the anode soon after the electrolysis, the migration from the cathode to the anode side also occurred early, thus leading to more severe influence on the NH_4_^+^ concentration at the cathode side. (2) The experiments were conducted with different concentrations of HCl solutions (0.01, 0.05, and 0.1 m) containing 2 ppm NH_4_^+^. Aqueous HCl solutions with concentrations above 0.1 m were not used since high concentrations of HCl can affect the Berthelot reaction, which is the basis for the indophenol blue method,^[^ ^9^ ^]^ thus would adversely influence the accuracy of our measurement of NH_4_^+^. We primarily used 0.1 m HCl for our study of the migration and oxidation of NH_4_^+^, as it is the most commonly applied acidic electrolyte in ENRR, allowing for our results to be more directly compared with those reported in the prior literature. As displayed in Figure 4b, more NH_4_^+^ was oxidized with increasing concentration of HCl, which was ascribed to the fact that a larger amount of HClO was produced with higher concentration of HCl. (3) Experiments with different types of solutions (0.1 m NaCl and KCl) containing Cl^−^ and 2 ppm NH_4_^+^ were performed. The altering trends of NH_4_^+^ in both cells were similar to that of 0.1 m HCl (Figure 4c). These results further confirmed the effect of Cl^−^ on the oxidation and diffusion of NH_4_^+^. Furthermore, the same phenomenon was also observed in Br^−^‐based solutions (Figure S15, Supporting Information). However, F^−^‐containing anolytes have only a minor impact on the NH_4_^+^ concentration at the cathode side, as the oxidation potential of F^−^ is 2.87 V (vs RHE), substantially higher than the onset potential of OER. A consequence of this difficulty with oxidation of F^−^ at the anode is that oxidation of the migrated NH_4_^+^ is also limited, due to a lack of oxidized F^−^. (4) Long‐term electrolysis experiments were carried out to evaluate the diffusion and oxidation of NH_4_^+^. It was found that the NH_4_^+^ in the catholyte cell gradually decreased and after 100 h of electrolysis, there was scarcely any NH_4_^+^ left in the cathode cell due to the continuous diffusion and subsequently rapid oxidation in the anolyte (Figure 4d). Long‐term experiments were also conducted at open circuit (Figure S16, Supporting Information), the results of which showed that the NH_4_^+^ in the cathode and anode cell eventually reached equilibrium (the concentration of NH_4_^+^ in the cathode and anode compartment was 1.05 and 0.93 ppm, respectively). The above results indicate that the remarkable influence on the quantification of ENRR is caused by the migration and oxidation of NH_4_^+^ between the two compartments, especially for long duration electrolysis operation.

**Figure 5:**
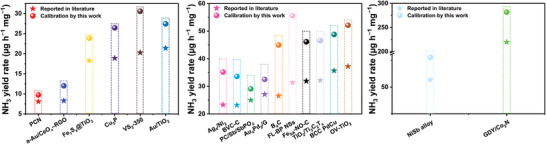
Comparison of ammonia yield rates reported in literature and the calibrated values based on this work.^[^ ^32^, ^33^, ^34^, ^35^, ^36^, ^37^, ^38^, ^39^, ^40^, ^41^, ^42^, ^43^, ^44^, ^45^, ^46^, ^47^, ^48^, ^49^ ^]^

To date, as reported in prior literature, the concentrations of ammonia produced by ENRR are still at the ppm level. Our findings highlight the marked Cl^−^ containing anolyte effect, which was however neglected in previous works. Namely, the generation of HClO by anodic oxidation of Cl^−^‐based electrolytes and the subsequent oxidation of the diffused NH_4_^+^ from the cathode compartment, will intensify the migration of NH_4_^+^ from cathode to the anode side and cause severe errors in the quantification of ammonia in the ENRR. Indeed, in view of our study, we conclude that solutions containing Cl^−^ ions are not suitable for use as anolytes. With the benefit of our findings, we have attempted to calibrate recently reported ammonia yield rates, based on our results (**Figure** **5**). It can be found that the error caused by the anolyte effect has a significant negative influence on the experimental results. The ammonia yield rates can be underestimated by up to 43.56%. Especially, if a single‐chamber reactor is used with Cl^−^ containing solutions as the electrolyte, the ammonia produced during the electrolysis will be rapidly oxidized, thus little or no ammonia can be detected after the electrolysis reaction. This can undoubtedly lead to misleading information on the performance of ENRR catalytic systems.

Besides the Cl^−^ containing solutions discussed above, other solutions that contain or produce strongly oxidizing substances during electrolysis (such as Br^−^‐based solutions) are also unsuitable anolytes. In addition to electrochemical nitrogen reduction to ammonia, other emerging electrochemical ammonia synthesis technologies (e.g., electrocatalytic nitrate reduction to ammonia, electrocatalytic NO*_x_* reduction to ammonia, etc.) have attracted significant interest recently. The concentrations of ammonia produced from these processes are usually much higher than that from ENRR. Accordingly, the migration and oxidation of ammonium can be more severe and cause a substantial influence on the determination of faradaic efficiency for ammonia. Therefore, we should rigorously reassess the ammonia yields in these systems.

## 3 Conclusion

In conclusion, we report the severe influence of oxidizing (Cl^−^ and Br^−^‐based) anolytes on the ENRR. With Cl^−^ containing anodic electrolytes, Cl^−^ oxidation to Cl_2_ will occur during ENRR. The formation of HClO from Cl_2_ dissolution can oxidize the diffused NH_4_^+^ from the cathode cell, which in turn intensifies the migration of NH_4_^+^ from the cathode to the anode cell, leading to large errors in the quantification of ammonia in ENRR. Such a finding can also be applicable to other electrochemical ammonia synthesis technologies, thus providing a guideline for rational design and use of anolytes during electrochemical ammonia production.

## Conflict of Interest

The authors declare no conflict of interest.

## Supporting information

Supporting Information

## Data Availability

The data that support the findings of this study are available from the corresponding author upon reasonable request.
