# Supplementary material for: Rigorous Assessment of Cl−‐Based Anolytes on Electrochemical Ammonia Synthesis
Source: Adv Sci (Weinh). 2022 Oct 17;9(33):2204205. doi: 10.1002/advs.202204205 (PMC9685447; doi:10.1002/advs.202204205)
Supplement: Supplementary file 1 — Supporting Information [file ADVS-9-2204205-s001.pdf]

Rigorous assessment of Cl^{-}-based anolytes on electrochemical ammonia synthesis

Zengxiang Lv[1], *Leiduan Hao*[1], *Zhibo Yao*[1], *Weixiang Li*[1], *Alex W. Robertson*[2], *Zhenyu Sun**[1]

[1] State Key Laboratory of Organic-Inorganic Composites College of Chemical Engineering, Beijing University of Chemical Technology, Beijing10029, P. R. China.

[2] Department of Physics, University of Warwick, Coventry CV4 7AL, U. K.

*Corresponding authors: sunzy@mail.buct.edu.cn.

Experimental procedures

1. Chemicals and materials

All chemicals used in this work were of analytical grade and used without further treatments. Sodium sulfate anhydrous (Na_2SO_4, ≥99.0%), Potassium hydroxide (KOH, ≥99.999%), Ammonium chloride (NH_4Cl, ≥99.99%), Sodium nitroferricyanide dihydrate ($C_5Fe_6Na_2O.2H_2O$, ≥99%), Sodium hypochlorite solution (NaClO, active chlorine 6~14%), hydrogen peroxide (H_2O_2, ≥30%) phosphate buffer solution (pH=6.5), sodium chloride (NaCl, ≥99.5%) were all purchased from Aladdin. Potassium chloride (KCl, ≥99.99%), Potassium sodium tartrate ($KNaC_4H_4O_6.4H_2O$, ≥99%), *N*,*N*-diethyl-1,4-phenylenediamine ($C_{10}H_{16}N_2$, ≥98%) were obtained from Macklin. Sodium hydroxide (NaOH, ≥98%), sodium citrate tribasic dihydrate ($C_6H_5Na_3O_7$, ≥99%), salicylic acid ($C_7H_6O_3$) were provided by Sigma-Aldrich. Ethanol,(C_2H_6O, ≥99%), hydrochloric acid (HCl, 36~38%), sulfuric acid (H_2SO_4, 95～98%) were provided by Beijing Chemical Works. Carbon paper was bought from Japan Toray. Nafion 115 and 117 membranes were purchased from Alfa Aesar. Nafion 211 membrane was brought from Suzhou Sinero Technology Co., Ltd. Nessler reagent was purchased from Shanghai yuanye Bio-Technology Co., Ltd. The ultrapure water (18.2 MΩ cm^{-1}) for all experiment was prepared by a purification system. Argon gas (99.999% purity) was supplied by Beijing Haipu Gas Co., Ltd.

2. Electrochemical measurements

The electrochemical analysis was performed with a standard three-electrode system via a DH-7000 (Jiangsu Donghua) electrochemical workstation in a typical H-type cell. The Nafion membranes were pretreated by heating in 5% H_2O_2 aqueous solution and 0.5 M H_2SO_4 at 80 °C for 1 h, respectively. Before use the Nafion membrane was immersed in deionized water under ambient conditions for 30 min and wash with deionized water. The carbon paper was pretreated by 30 min of ultrasonication in absolute ethyl alcohol and deionized water, respectively. A Pt wire, an Ag/AgCl (saturated KCl solution), and pure carbon paper were used as the counter electrode, reference electrode and working electrode, respectively. All potentials in this study were converted to RHE with the equation: $E_{RHE} = E_{Ag/AgCl} + 0.05916 \times pH + 0.197$. The electrochemical experiments were conducted in Ar-saturated solution at room temperature and atmospheric pressure. A flow of ultra-pure Argon gas (99.999%) with a rate of 25 mL min^{-1} was purged for 20 min to remove residual air in the reservoir. During the electrolysis, the Ar was continuously purged into the anode and cathode cell at a flow rate of 25 mL min^{-1}. In the meantime, magnetic stirring was performed in the cathodic compartment and anodic compartment at a rate of 400 rpm. After electrolysis, the NH_4^+ was quantitatively determined by the indophenol blue method, Nessler reagent method and ion chromatography (IC).

3. Determination of ammonia

Indophenol blue method: NH_4^+ concentration analysis was conducted using the indophenol blue method. Firstly, 2 mL of electrolyte was taken from the anode and cathode chamber, and then dispersed into 2 mL of 1 M NaOH solution consisting of salicylic acid (5 wt.%) and sodium citrate (5 wt.%), followed by the addition of 1 mL NaClO (0.05 M) and 0.2 mL $C_5FeN_6Na_2O$ (1 wt.%) and homogeneously mixed. The absorption spectrum was carried out using a UV-vis spectrophotometer

after standing for 2 h at room temperature. The absorbance at the wavelength of 655 nm was used to determine the NH_4^+ concentration by PERSEE TU-1950 UV-VIS spectrophotometer.

Nessler reagent method: Specifically, 2 mL tested solution was first added into a reagent bottle. Then 2 mL of 1 M KOH solution and 1 mL of 0.2 M potassium sodium tartrate ($KNaC_4H_4O_6$) solution were added into the bottle, respectively. After mixing thoroughly, 0.5 mL of Nessler reagent was added and kept for 1 h at 25 °C. Finally, the absorbance of the sample was measured through a UV–vis spectrophotometer from 390 to 550 nm. The absorbance at the wavelength of 420 nm was used to determine the NH_4^+ concentration.

Ion chromatography method: The ammonia was also detected by Ion chromatography (Thermo Scientific™ Dionex™ Aquion™). Gas products (N_2O, N_2, and H_2) were quantified on a Gas chromatography (GC 2014 SHIMADZU) equipped with Porapak N and Molsieve 5 Å columns leading to a thermal conductivity detector (TCD).

4. Determination of hypochlorous acid

The content of hypochlorous acid was measured by revised *N*,*N*-diethyl-1,4-phenylenediamine (DPD) method. Briefly, 2 mL of teste electrolyte was pipetted and mixed with 2 mL of Phosphate buffer solution (pH = 6.5), followed by the addition of 1 mL of 6 g/L DPD ethanol solution. Then homogeneously mixed and completed the test within 5 minutes. The HClO amounts were determined based on the absorbance at 513 nm.

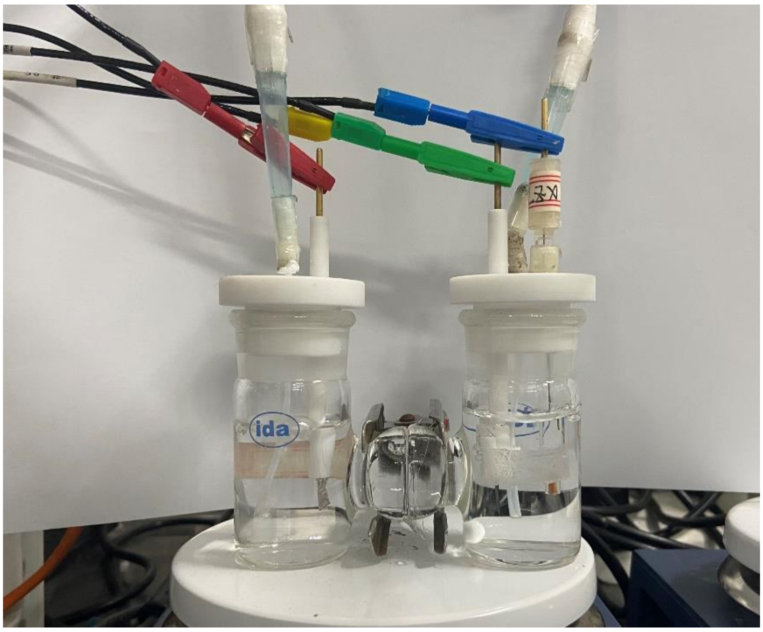

Figure S1. The photograph of a typical H-type cell separated by Nafion membrane for this study.

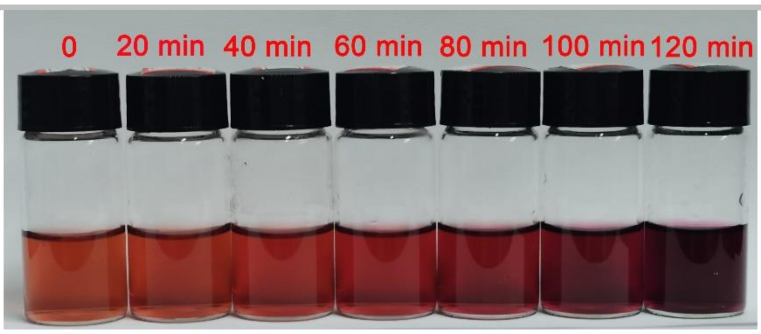

Figure S2. The images of HClO via DPD assay in the anode chamber at different times during electrolysis at −0.4 V (*vs.* RHE) using 0.1 M HCl as an electrolyte.

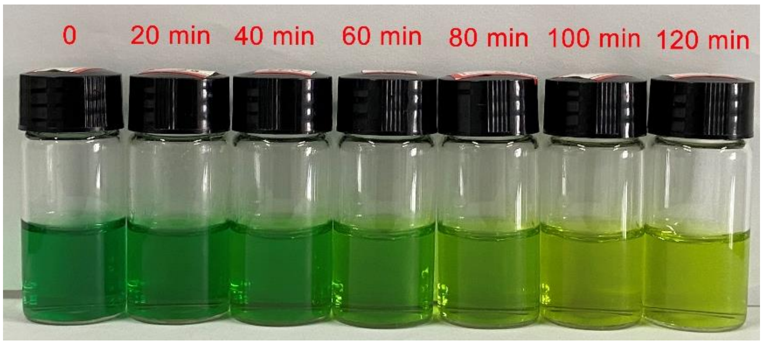

Figure S3. The images of NH_4^+ measured through an indophenol blue method in anode chamber at different times during electrolysis at −0.4 V (*vs.* RHE) using 0.1 M HCl containing 2 ppm NH_4^+ as both anode and cathode electrolytes.

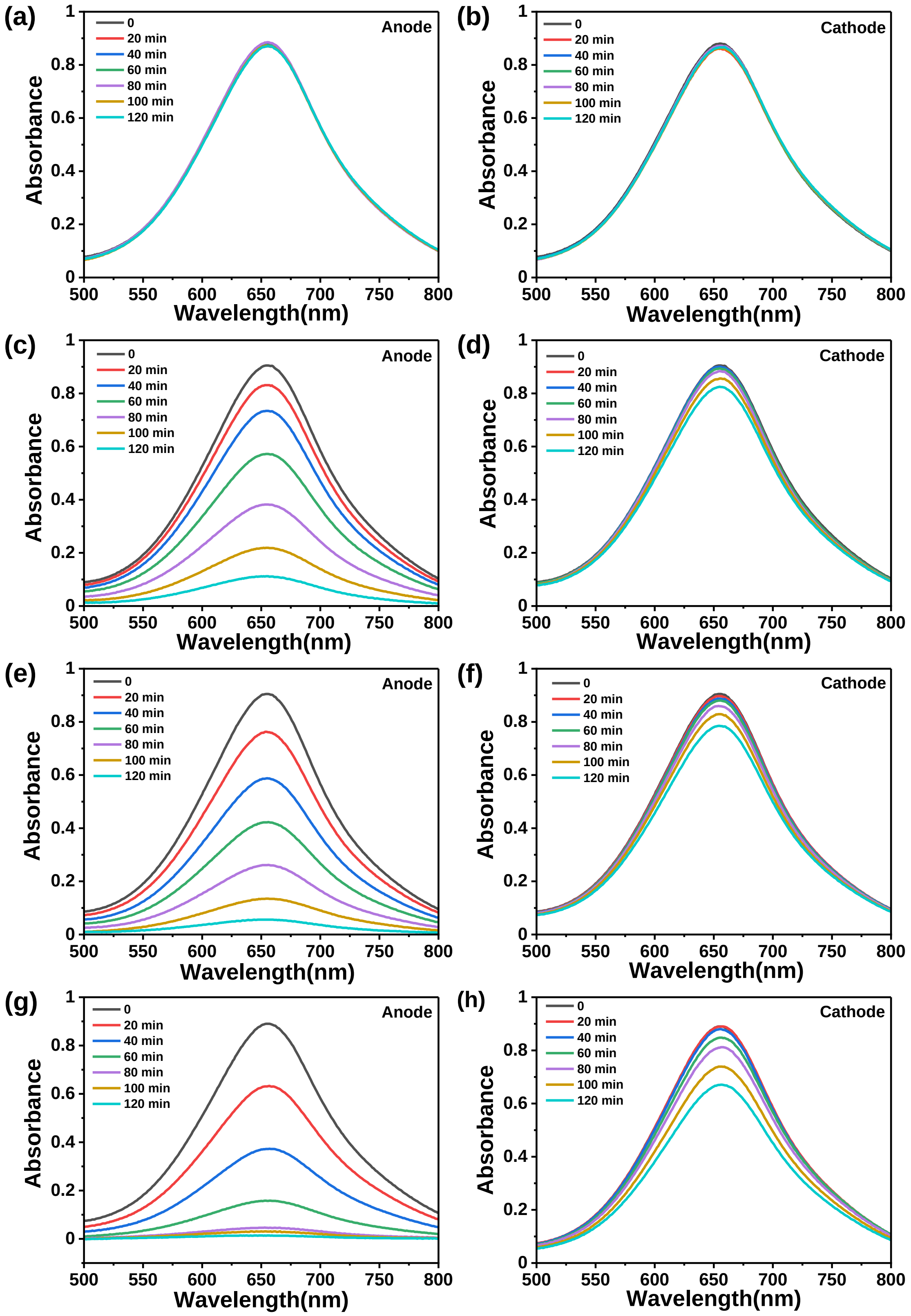

Figure S4. The UV-vis spectra of electrolytes in anode cell (a) and cathode cell (b) at different moments under an open circuit potential for 0.1 M HCl solution containing 2 ppm NH_4^+ as both anode and cathode electrolytes. (c) and (d) correspond to the UV-vis spectra of the anode cell and cathode cell at −0.1 V (*vs*. RHE). (e) and (f) correspond to

the UV-vis spectra of the anode cell and cathode cell at −0.2 V (*vs.* RHE). (g) and (h) correspond to the UV-vis spectra of the anode cell and cathode cell at −0.4 V (*vs.* RHE), respectively.

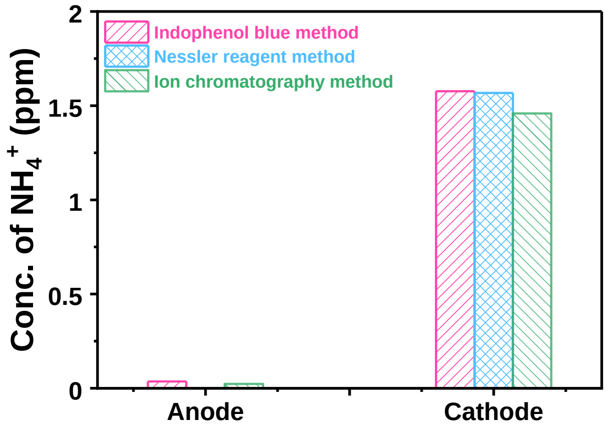

Figure S5. The NH_4^+ concentration of the anode cell and cathode cell after 2 hours of reaction under −0.4 V (*vs.* RHE) measured by different methods.

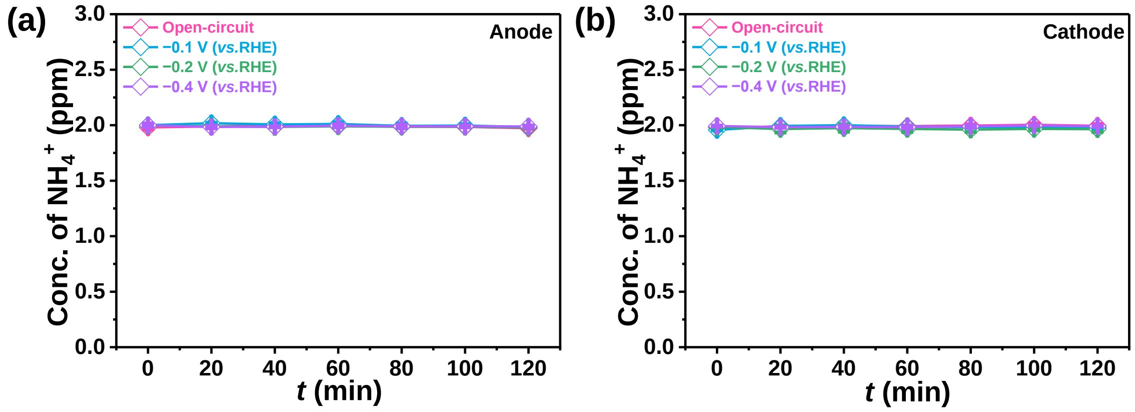

Figure S6. The variation trend of NH_4^+ in (a) anolyte and (b) catholyte with time at an open circuit and various potentials in 0.1 M Na_2SO_4.

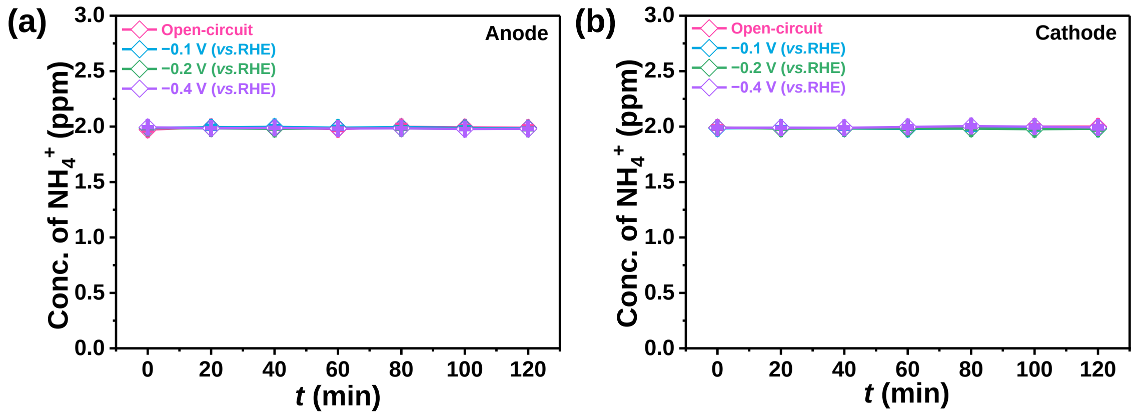

Figure S7. The variation trend of NH_4^+ in the (a) anolyte and (b) catholyte with time at an open circuit and various potentials in 0.1 M KOH.

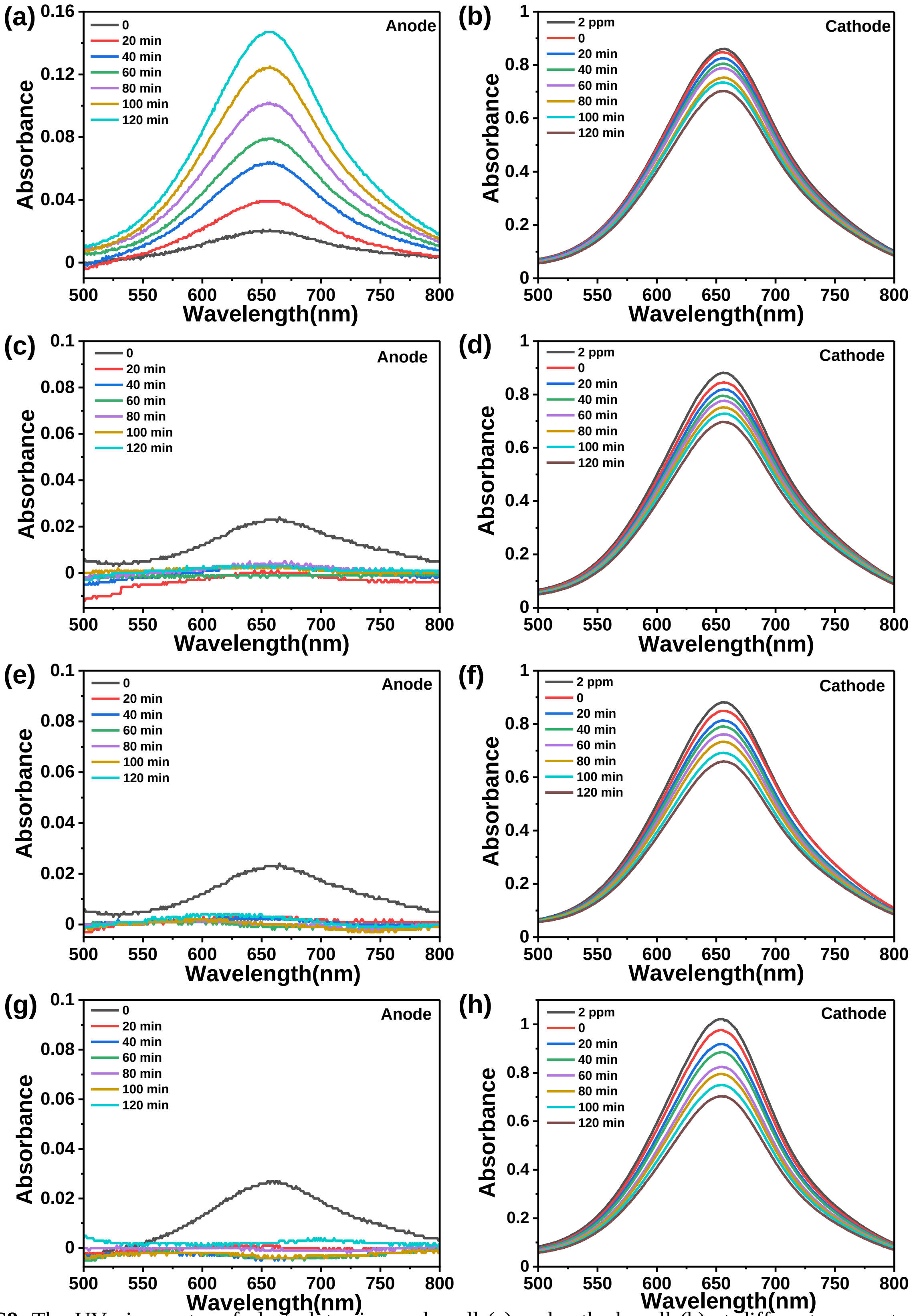

Figure S8. The UV-vis spectra of electrolytes in anode cell (a) and cathode cell (b) at different moments under an open circuit potential for 0.1 M HCl without NH_4^+ as the analyte and 0.1 M HCl containing 2 ppm NH_4^+ as the catholyte. (c) and (d) correspond to the UV-vis spectra of the analyte and catholyte at −0.1 V (*vs*. RHE). (e) and (f) correspond to the UV-vis spectra of the analyte and catholyte at −0.2 V (*vs*. RHE). (g) and (h) correspond to the UV-vis spectra of the analyte and catholyte at −0.4 V (*vs*. RHE), respectively.

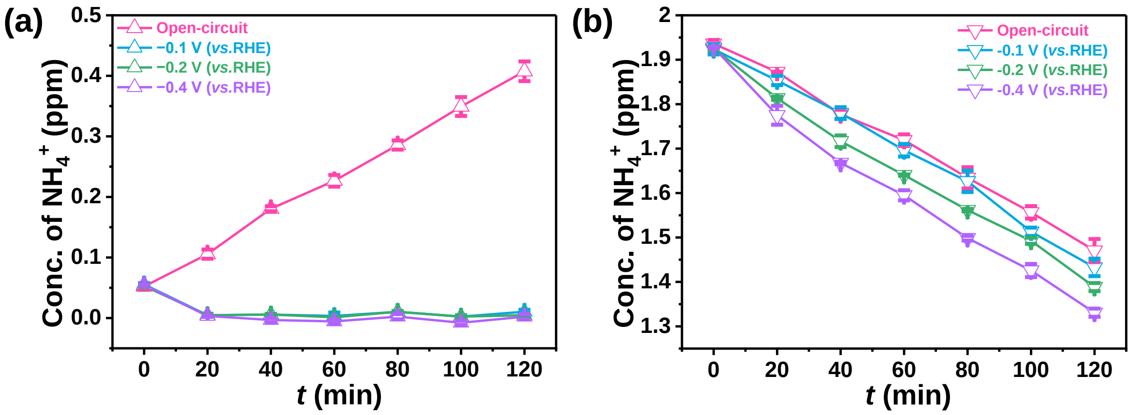

Figure S9. The variation of NH_4^+ in anode solution (a) and cathode solution (b) with time at an open circuit and various potentials in 0.1 M HCl using Nafion 115 as a proton exchange membrane.

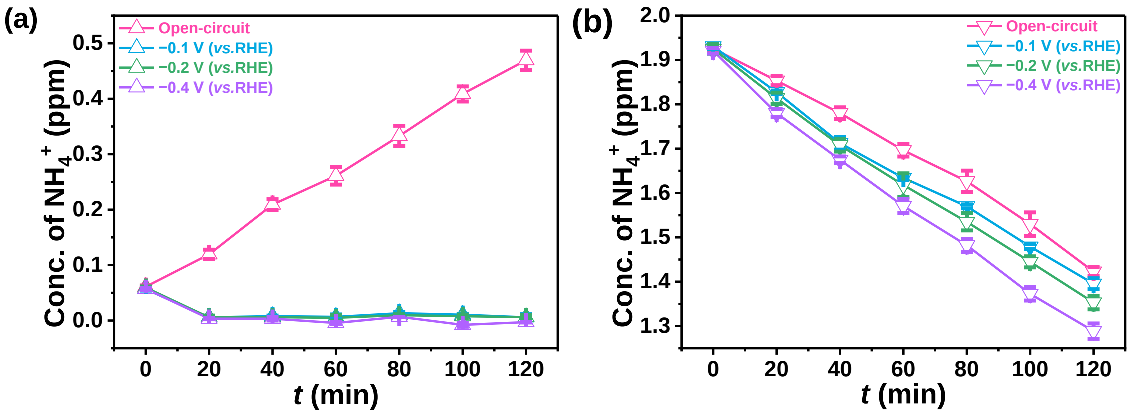

Figure S10. The variation of NH_4^+ in anode solution (a) and cathode solution (b) with time at an open circuit and various potentials in 0.1 M HCl using Nafion 211 as a proton exchange membrane.

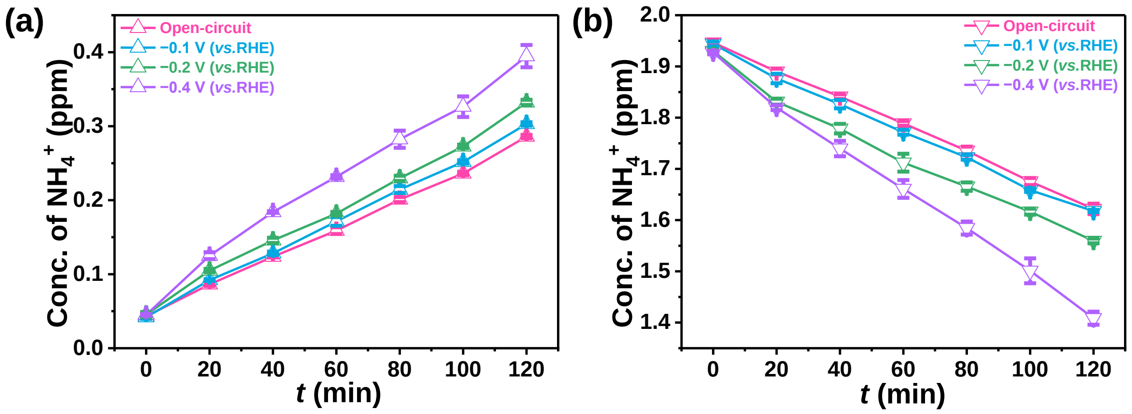

Figure S11. The variation of NH_4^+ in (a) anolyte and (b) catholyte as a function of time at an open circuit and various potentials in 0.1 M Na_2SO_4.

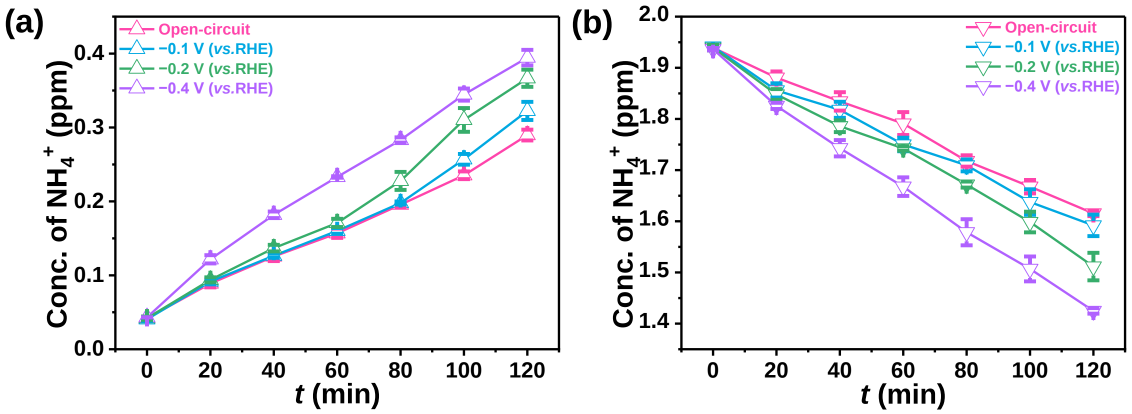

Figure S12. The variation of NH_4^+ in anode solution (a) and cathode solution (b) with time at an open circuit and various potentials in 0.1 M KOH.

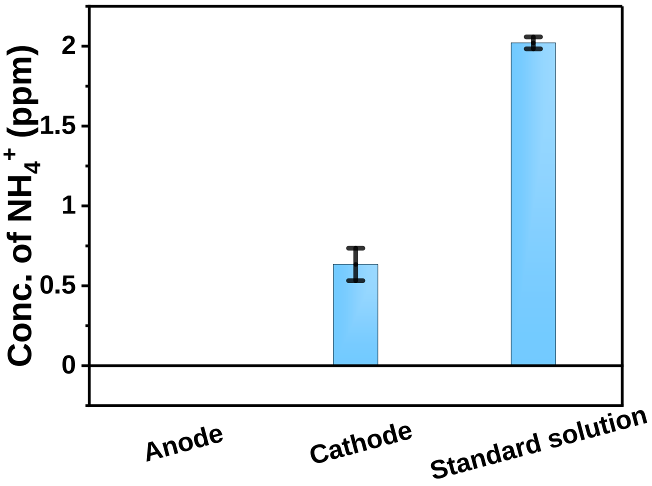

Figure S13. The concentration of NH_4^+ after electrolysis at −0.4 V (*vs*. RHE) for 2 h by using a flow cell.

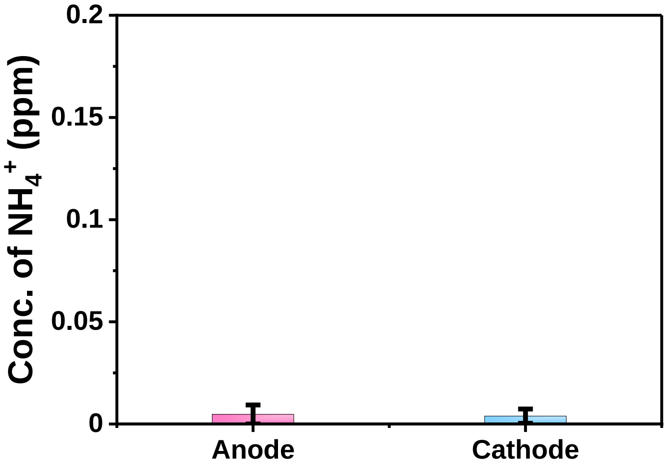

Figure S14. Ammonia in the anode cell and cathode cell after 2 h of reaction using a solution without ammonia as the electrolyte.

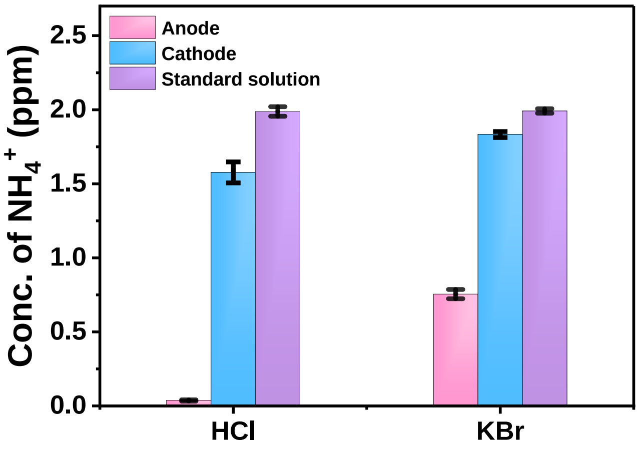

Figure S15. Comparison of NH_4^+ concentration after 2 h of electrolysis at −0.4 V (*vs.* RHE) with Cl^- and Br^- based solutions containing 2 ppm NH_4^+.

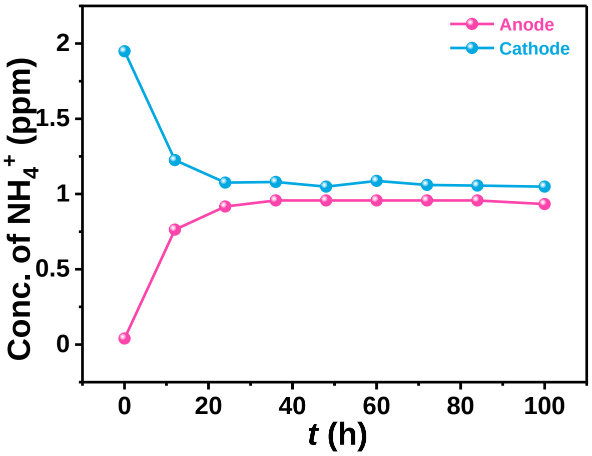

Figure S16. The trend of NH_4^+ in anode and cathode chamber during 100 h at an open circuit potential for 0.1 M HCl without NH_4^+ as anolyte and 0.1 M HCl containing 2 ppm NH_4^+ as catholyte.

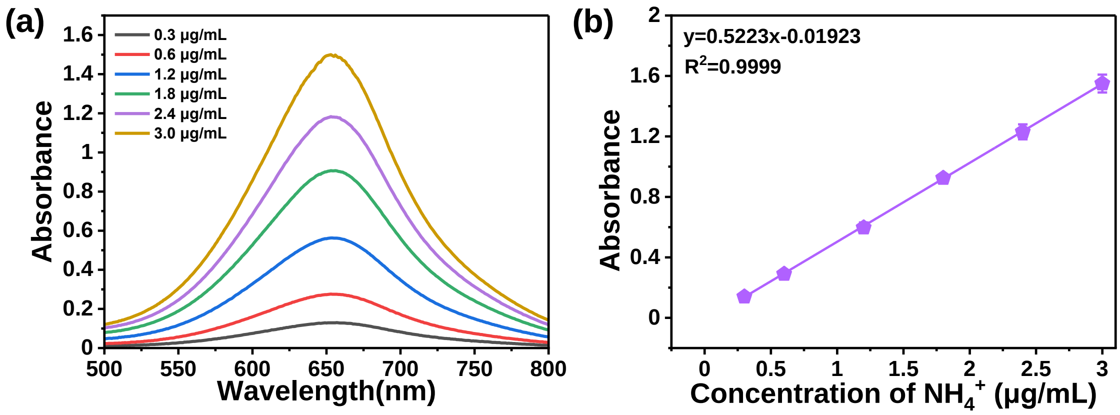

Figure S17. (a) UV-vis absorption spectra of indophenol assays with NH_4Cl in 0.1 M HCl after being incubated for 2 h at ambient conditions. (b) Calibration curve used for the calculation of NH_4^+ concentrations.

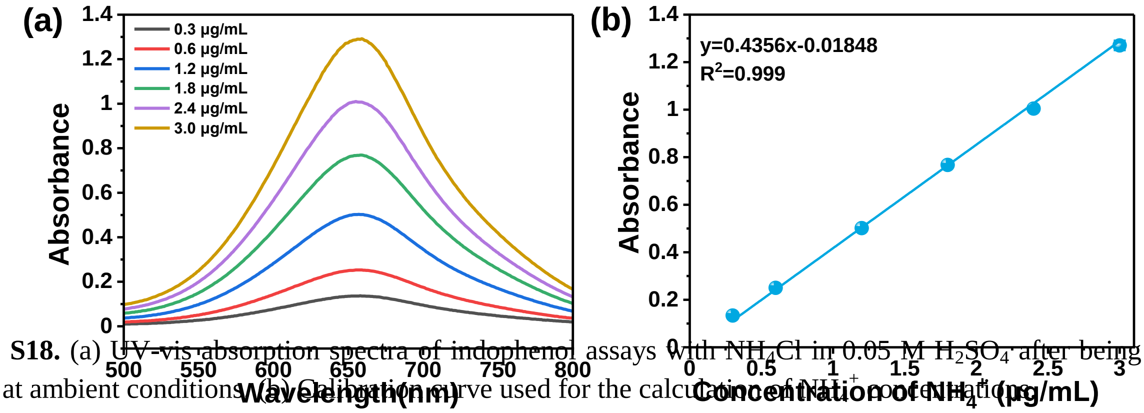

Figure S18. (a) UV-vis absorption spectra of indophenol assays with NH_4Cl in 0.05 M H_2SO_4 after being incubated for 2 h at ambient conditions. (b) Calibration curve used for the calculation of NH_4^+ concentrations.

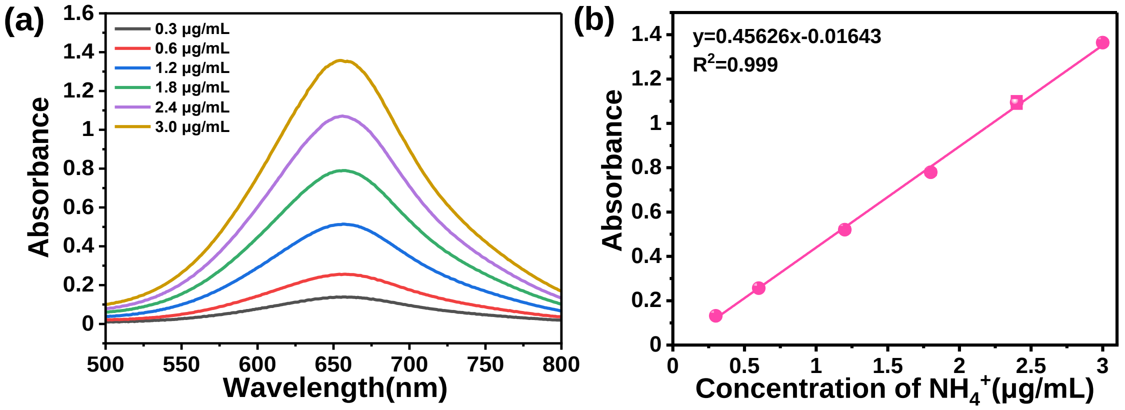

Figure S19. (a) UV-vis absorption spectra of indophenol assays with NH_4Cl in 0.1 M Na_2SO_4 after being incubated for 2 h at ambient conditions. (b) Calibration curve used for the calculation of NH_4^+ concentrations.

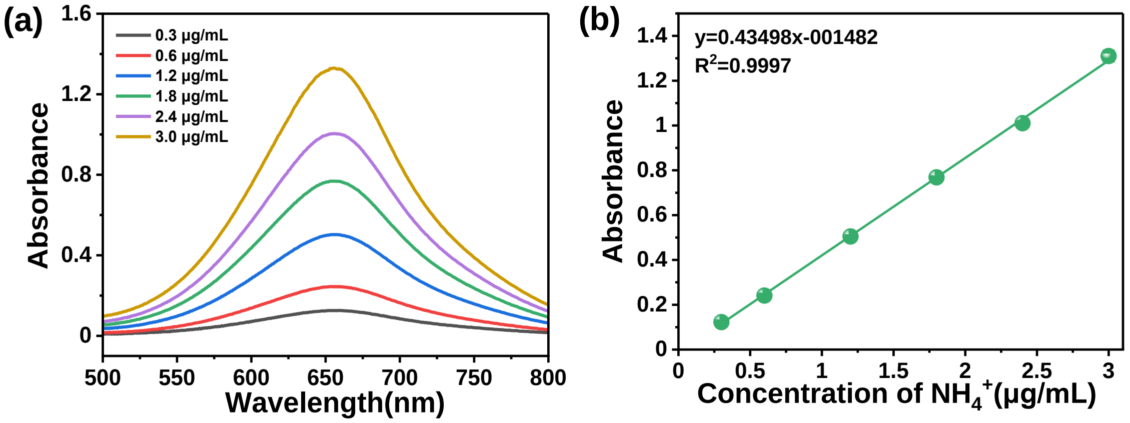

Figure S20. (a) UV-vis absorption spectra of indophenol assays with NH_4Cl in 0.1 M KOH after being incubated for 2 h at ambient conditions. (b) Calibration curve used for the calculation of NH_4^+ concentrations.

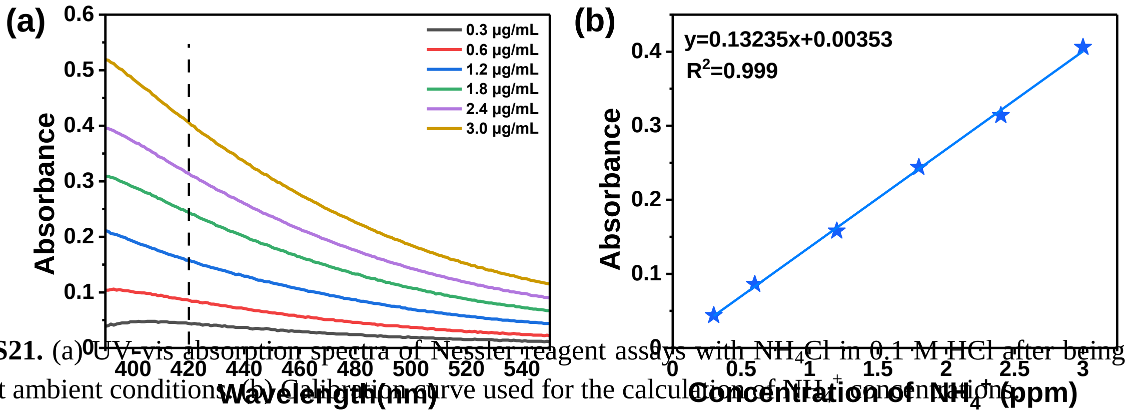

Figure S21. (a) UV-vis absorption spectra of Nessler reagent assays with NH_4Cl in 0.1 M HCl after being incubated for 1 h at ambient conditions. (b) Calibration curve used for the calculation of NH_4^+ concentrations.

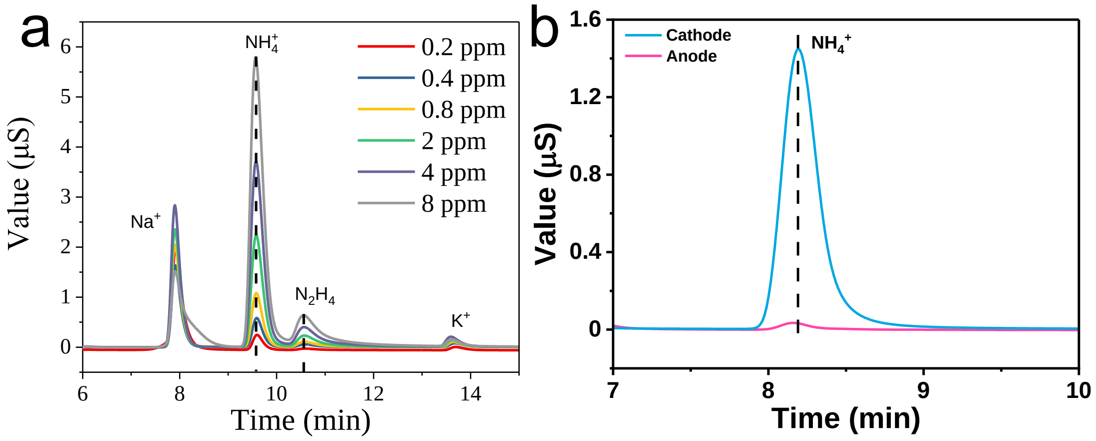

Figure S22. (a) Ammonium standard calibration by IC with coefficient of determination R^2 = 0.99974. (b) Ammonia concentration of cathode cell and anode cell after 2 h of electrolysis under −0.4 V (*vs*. RHE).

Table S1. The basic information of the membrane used in this work. The data was supplied by DuPont China Holding Co., Ltd.

Type	Thickness (μm)	Density (g/m^2)	Electrical conductivity (S/cm)	Exchange capacity (meq/g)
N117	183	360	0.083	0.89
N115	127	250	0.083	0.89
N211	25.4	50	0.083	0.95~1.01
